# Pigmented villonodular synovitis does not influence the outcome following cementless total hip arthroplasty using ceramic-on-ceramic articulation: a case-control study with middle-term follow-up

**DOI:** 10.1186/s13018-018-0996-6

**Published:** 2018-11-20

**Authors:** Chi Xu, Heng Guo, Kerri L. Bell, Feng-Chih Kuo, Ji-Ying Chen

**Affiliations:** 1 Department of Orthopaedic Surgery, General Hospital of People’s Liberation Army, No.28 Fuxing Road, Haidian District, Beijing, 100853 China; 2 Department of Orthopaedic Surgery, Beijing Mentougou District Hosptial, Beijing, China; 3 The Rothman Institute at Thomas Jefferson University, Philadelphia, PA USA; 4 Department of Orthopaedic Surgery, Kaohsiung Chang Gung Memorial Hospital, and Chang Gung University, College of Medicine, No. 123, Dapi Rd., Niaosong Dist, Kaohsiung, 833 Taiwan

**Keywords:** Synovitis, pigmented villonodular, Total hip arthroplasty, Ceramic-on-ceramic, Cementless

## Abstract

**Background:**

Pigmented villonodular synovitis (PVNS) is a relatively rare, locally aggressive, and potentially recurrent synovial disease of large joints. The purpose of this study was to investigate (1) the disease recurrence rate and (2) the treatment outcomes including Harris hip scores, complications, and revision following cementless total hip arthroplasty (THA) with ceramic-on-ceramic (CoC) articulation in patients with PVNS.

**Methods:**

Twenty-two patients (14 females and 8 males) with histologically confirmed PVNS underwent cementless THA using CoC bearings between 2000 and 2013. Three patients with less than 5-year follow-up were excluded. The mean age was 35.2 years (range, 22–58 years) with a mean follow-up of 8.6 years (range, 6.9–10.8 years). A control group was matched in a 2:1 ratio with the PVNS group for age, sex, body mass index (BMI), year of surgery, and American Society of Anesthesiologists score (ASA). Postoperative outcome variables included disease recurrence, Harris Hip Scores (HHS) at the latest follow-up, complications (dislocation, squeaking, ceramic fracture), and any-cause revision. A Kaplan-Meier implant survivorship curve with 95% confidence interval (CI) of the two groups was generated.

**Results:**

No recurrence of PVNS was noted in the follow-up period. The HSS in the PVNS group was 92.6 ± 5.5, which was similar to the control group (93.4 ± 4.6, *p* = 0.584) at the last follow-up visit. No patients sustained dislocation, osteolysis, or any ceramic fracture within the study duration. One patient in the PVNS group had a complication of squeaking, but did not require revision. Another patient in the PVNS group underwent revision surgery due to aseptic loosening. There was no significant difference in revision rates between the two groups (*p* = 1.000). The implant survivorship free of any revision was 90.0% (95% CI, 73.2% to 100%) in the PVNS group and 92.5% (95% CI, 82.6% to 100%) in the control group at 10 years (*p* = 0.99).

**Conclusions:**

For young and active patients with end-stage PVNS of the hips, cementless THA using CoC bearing has similar functional outcome scores, a low complication rate, and similar implant survivorship compared to the control group.

## Background

Pigmented villonodular synovitis (PVNS) of the hip is a benign, but potentially locally aggressive and recurrent monoarticular disorder which typically affects large joints; 80% of the time it involves the knee, while hips are affected only 15% of the time [1]. It is an uncommon disorder, with an estimated incidence of 1.8 per million patients [2–4]. It is characterized by a proliferation of synovial villi and nodules within joint spaces, bursa, or tendon sheaths [5]. As synovial hyperplasia pervades the hip, it causes discomfort by narrowing the joint space, leading to sharp hip pain [3]. A radical synovectomy, whether via arthroscopy or an open surgical approach, has been utilized as a treatment for hips with mild cartilage degeneration. However, in cases demonstrating severe end-stage arthritis due to disease progression, total hip arthroplasty (THA) plus radical synovectomy is the preferred treatment choice [6].

PVNS of the hip commonly affects patients in their third or fourth decade of life [7]. A recent study reported that complications and revision rates following THA are high in patients with PVNS due to the youth of patients and the utilization of conventional polyethylene [8]. Given the likelihood of requiring revision surgery for younger patients undergoing THA, the concern of implant survivorship, wear performance, and functionality should be considered when selecting a fixation technique and a bearing surface. As one of the most popular combinations, cementless implants with ceramic-on-ceramic (CoC) articulation have shown potential benefits in young patients, as they have low wear rates with excellent clinical outcomes [9–13]. Although several studies have suggested acceptable outcomes with a low recurrence rate following THA in PVNS patients during the long-term follow-up [1, 6, 8, 14], there is a paucity of data focusing on cementless implants using CoC articulation in these patients. Moreover, these previous studies were limited by small sample sizes and lacked a control group in their evaluation of outcomes.

Therefore, the purpose of this study was to investigate the disease recurrence rate and the treatment outcomes including Harris Hip Scores, complications, and revision rates following cementless THA with CoC articulation in patients with PVNS.

## Materials and methods

### Study cohort

After the Institutional Review Board approval, we retrospectively reviewed 22 patients with a diagnosis of PVNS of the hip who underwent cementless THA with CoC articulation between 2000 and 2013. The diagnosis of diffuse PVNS was made for all patients by histological confirmation in accordance with Jaffé’s classification [15]. Three patients with follow-up less than 5 years were excluded. In all, 19 patients with a mean follow-up of 8.6 years (range, 6.9 to 10.8 years) were enrolled in this study (Table 1). There were 7 males and 12 females with a mean age of 35.2 (range, 22 to 58) years.

**Table 1:** The patients with pigmented villonodular synovitis of the hip

Case	Age	Gender	Duration of symptoms (years)	Surgery before THA	Cup	Stem	FU (years)	Complications
1	28	Male	1	N/A	Pinnacle	Corail	8.6	–
2	26	Female	1.3	N/A	Betacup	Corail	9.3	Aseptic loosening
3	39	Female	2.4	N/A	Combicup	Ribbed	7.6	–
4	35	Female	3	Arthroscopic synovectomy	Pinnacle	Corail	8.8	–
5	53	Male	2.7	N/A	Betacup	Ribbed	10.6	–
6	24	Female	2	N/A	Combicup	Ribbed	8.5	–
7	40	Male	2.3	Internal fixation	Betacup	Ribbed	10.3	Squeaking
8	48	Female	2	N/A	Pinnacle	Corail	7.4	–
9	26	Male	4	N/A	Betacup	Ribbed	7.9	–
10	30	Female	6.2	N/A	Betacup	LCU	7.7	–
11	32	Female	3.8	Arthroscopic synovectomy	Pinnacle	Corail	8.3	–
12	58	Female	2.5	N/A	Pinnacle	Corail	7.7	–
13	28	Female	3	N/A	Combicup	LCU	6.9	–
14	47	Male	1.2	N/A	Combicup	Ribbed	10.8	–
15	35	Male	4.2	N/A	Pinnacle	Corail	7.9	–
16	22	Female	1.8	Arthroscopic synovectomy	Pinnacle	Corail	9.1	–
17	29	Female	2.8	Arthroscopic synovectomy	Betacup	LCU	9.7	–
18	37	Female	4.3	N/A	Betacup	LCU	7.8	–
19	31	Male	4	Arthroscopic synovectomy	Combicup	LCU	9.5	–

*THA* total hip arthroplasty, *LCU* link classic uncemented, *FU* follow-up, *N*/*A* not applicable

These patients were matched with a control group of patients; the controls were patients diagnosed with either osteoarthritis of the hip or femoral head necrosis who underwent cementless THA using CoC articulation. The patients in the control group were matched with the patients in the study group for age (± 5 years), sex, body mass index (BMI, ± 1 kg/m^2^), year of surgery (within 1 year), and American Society of Anesthesiologists Score (ASA) (± 1) in a 2:1 ratio.

### Clinical and radiographic features of patients with PVNS before THA

The mean time from onset of symptoms to arthroplasty was 2.9 years (range, 1–6.2 years). All patients presented hip pain as the primary complaint. One patient, a 28-year-old woman (case 13), was diagnosed with Crowe Type III developmental hip dysplasia. Four patients had hip trauma before THA, while another patient had undergone internal fixation with cannulated screws for a fracture of the femoral neck. Five patients had undergone arthroscopic synovectomy before THA; the mean time from the last synovectomy to THA was 2.4 years (range, 0.8–3.6 years). A limitation of hip motion (ranging between 10 and 30°) was identified in eight patients. Average flexion was 91.1° (range, 35–130°). Elevation of both C-reactive protein (CRP) and erythrocyte sedimentation rate (ESR) was identified in two patients. The serum white blood count, CRP, and ESR were normal in the other 17 patients.

**Fig. 1:**
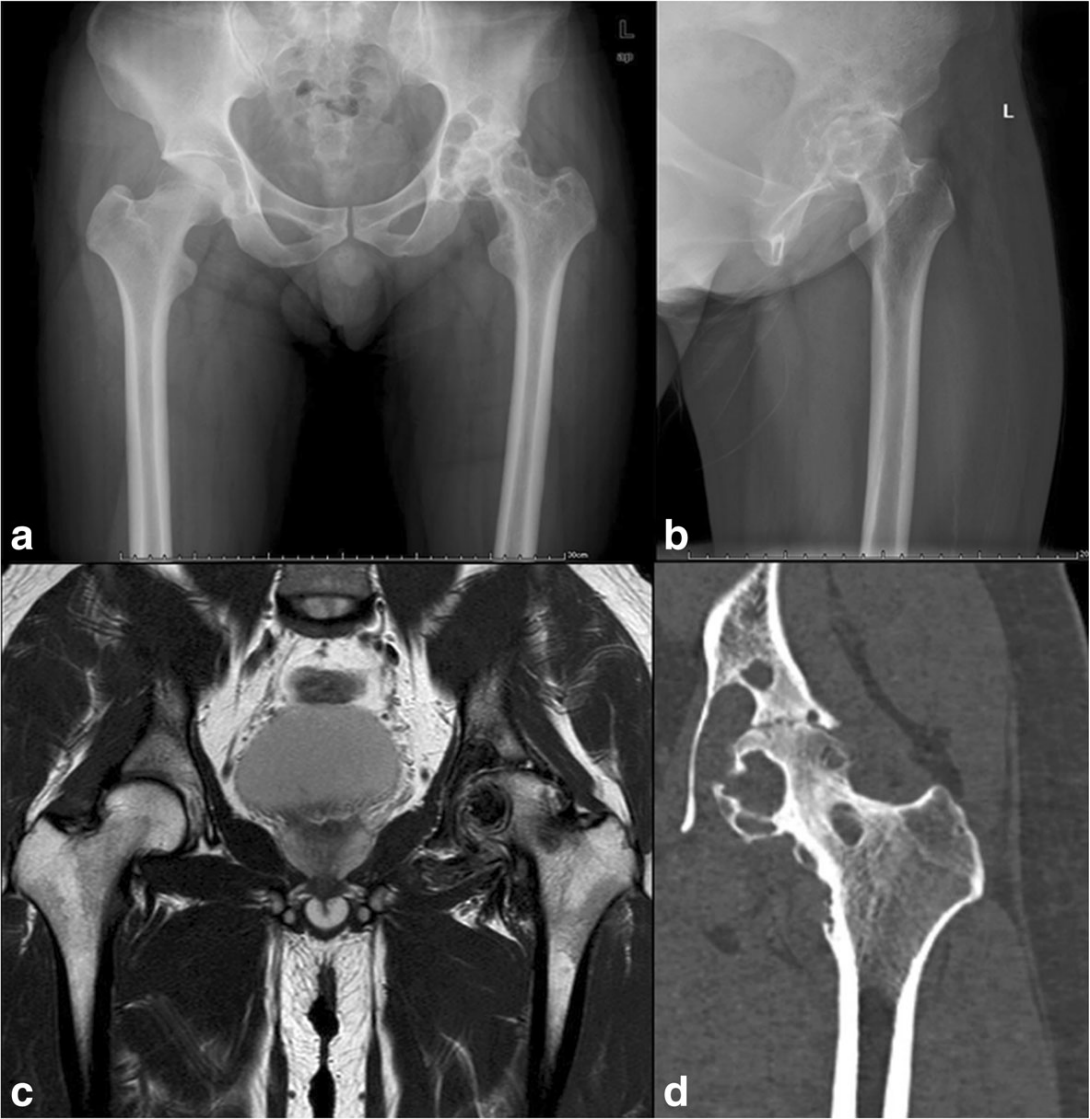
The typical appearance of PVNS as seen in radiography of the hip, magnetic resonance imaging (MRI), and computed tomography (CT). Anteroposterior radiograph of case 5 (**a**) and lateral radiograph of the case 16 (**b**) show erosions and cysts in both the femoral head and the acetabulum of left hip with nearly complete obliteration of the joint space. CT (**c**) and MRI (**d**) of case 14 show the characteristic cyst-like structure of the femoral head

Radiographs showed the typical characteristics of end-stage PVNS of the hip including cystic erosions and joint space narrowing (Fig. 1). Eight patients had the nearly complete disappearance of the joint space. Seven patients underwent preoperative magnetic resonance imaging, which showed the characteristic low signal intensity on the T2-weighted sequences and blooming artifact from the high hemosiderin on the gradient-echo sequences.

### Surgical treatment

An institutional standard protocol for total hip arthroplasty was performed for all cases by experienced arthroplasty surgeons. A posterolateral approach was utilized for all patients within the study. For patients with PVNS, complete radical excision of the diseased synovium was performed and pathologic synovial tissues were submitted for histologic evaluation. Removal of the femoral head and neck provided enough space between the pelvis and the femur for a synovectomy. The tissue contained within the cystic areas of the acetabulum and the femur was carefully removed. Cementless implants using ceramic-on-ceramic bearings were inserted in all patients. Pinnacle^®^ (DePuy, Warsaw, IN, USA) or Betacup^®^/Combicup^®^ (Link, Hamburg, Germany) were used for cup components, while Corail^®^ (DePuy, Warsaw, IN, USA) or Ribbed^®^/LCU^®^ Link Classic Uncemented (Link, Hamburg, Germany) were used for stem components.

The postoperative protocol included a drainage tube and prophylactic administration of antibiotics for 24 h. The patients were allowed partial weight-bearing with a walking aid after a postoperative radiographic evaluation on the first postoperative day, and then were allowed full weight-bearing at 6–8 weeks. The duration of prophylaxis for deep vein thrombosis was for 30 days.

### Clinical and radiologic evaluation

Hip function was evaluated using Harris Hip Scores (HHS) before THA and at the last follow-up of each patient [16]. Questionnaire responses regarding the need for walking aids and the ability to perform moderate and strenuous activities were also recorded. Radiographic loosening was defined in accordance with previously published studies: a loose acetabular component was defined if the cup had ≥ 2 mm migration, ≥ 5° changes in tilting, shedding of metal particles, or a continuous radiolucent line across all zones, as described by Delee and Charnley [17, 18]; a loose femoral stem was characterized by ≥ 5 mm progressive subsidence, pedestal formation, the shedding of metal particles, or a continuous radiolucent line around the stem, as defined by Gruen et al. [19]. Periprosthetic osteolysis was determined to be present if there were periprosthetic lesions ≥ 2 mm in diameter on follow-up radiographs [20]. Local recurrence of PVNS was determined by a series of radiological changes in adjacent bone (e.g., cystic erosions without calcification or sclerosis, demineralization of surrounding bone) [21]. All radiologic evaluations were performed by two experienced orthopedic surgeons. Complications related to CoC THA included infection, dislocation, squeaking, or ceramic fracture were recorded.

### Statistical analysis

Categorical variables were presented as frequencies and percentages, and continuous variables as means and standard deviations. The clinical characteristics between groups were compared with the use of the independent *t* test or the Mann-Whitney test for continuous variables and the chi-squared test or Fisher’s exact test for categorical variables. A Kaplan-Meier implant survival curve was generated with 95% confidence intervals (CI) at 10 years. The end point was set at the any-cause revision of CoC THA (e.g., aseptic loosening, infection, osteolysis, recurrent dislocation, ceramic fracture, or squeaking). A *p* value of < 0.05 was considered significant. All of the statistical analyses were performed with the statistical software packages R (http://www.R-project.org, The R Foundation) and EmpowerStats (http://www.empowerstats.com, X&Y Solution, Inc., Boston, MA).

## Results

### Cohort and specimen characteristics

**Fig. 2:**
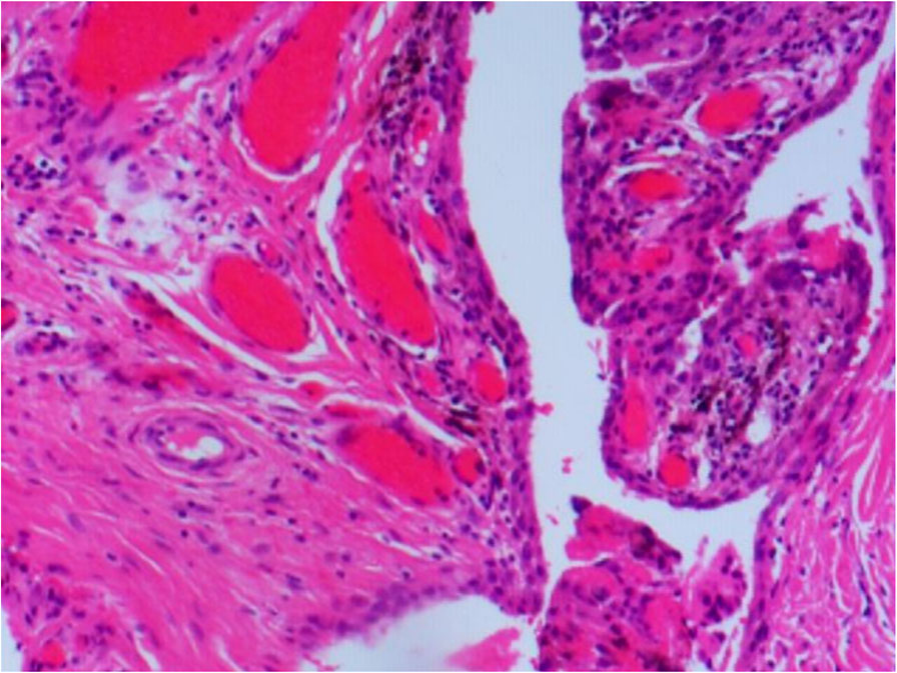
A photomicrograph of case 14 showing synovial hypertrophy and histiocyte proliferation with multinucleated giant cells. (hematoxylin and eosin, original magnification × 200)

Due to strict matching, the age, sex, BMI, and ASA scores were similar between the two groups (Table 2). Of the 38 controls, 27 were patients with osteonecrosis of the femoral head while 11 were diagnosed with osteoarthritis of the hip. The mean follow-up for the PVNS and the control group was 8.6 ± 1.1 years and 8.7 ± 1.3 years, respectively. The diagnosis of PVNS was made by pathological examination of all 19 patients. Macroscopically, the pathologic synovial tissues were brown or yellowish. Histologically, there were numerous synovial cell and multinucleated giant cells (Fig. 2). Hemosiderin-laden macrophages were found in 13 of 19 patients. We did not identify any malignant changes or atypical cytology.

**Table 2:** Patient characteristics and follow-up between the PVNS group and the control group

	PVNS (*n* = 19)	Controls (*n* = 38)	*p* value
Age (year), mean ± SD	35.2 ± 10.2	35.2 ± 10.0	1.000
Gender, *n* (%)			1.000
Female	12 (63.2%)	24 (63.2%)	
Male	7 (36.8%)	14 (36.8%)	
Body mass index (kg/m^2^), mean ± SD	24.6 ± 2.2	24.5 ± 1.9	0.975
American Society of Anesthesiologists score			1.000
1	14	28	
2	4	8	
3	1	2	
Diagnosis, *n* (%)			–
Osteonecrosis	0 (0%)	27 (71%)	
Osteoarthritis	0 (0%)	11 (29%)	
PVNS	19 (100%)	0 (0%)	
Follow-up (years), mean ± SD	8.7 ± 1.3	8.6 ± 1.1	0.902

*PVNS* pigmented villonodular synovitis, *SD* standard deviation

### PVNS and outcomes following THA

**Fig. 3:**
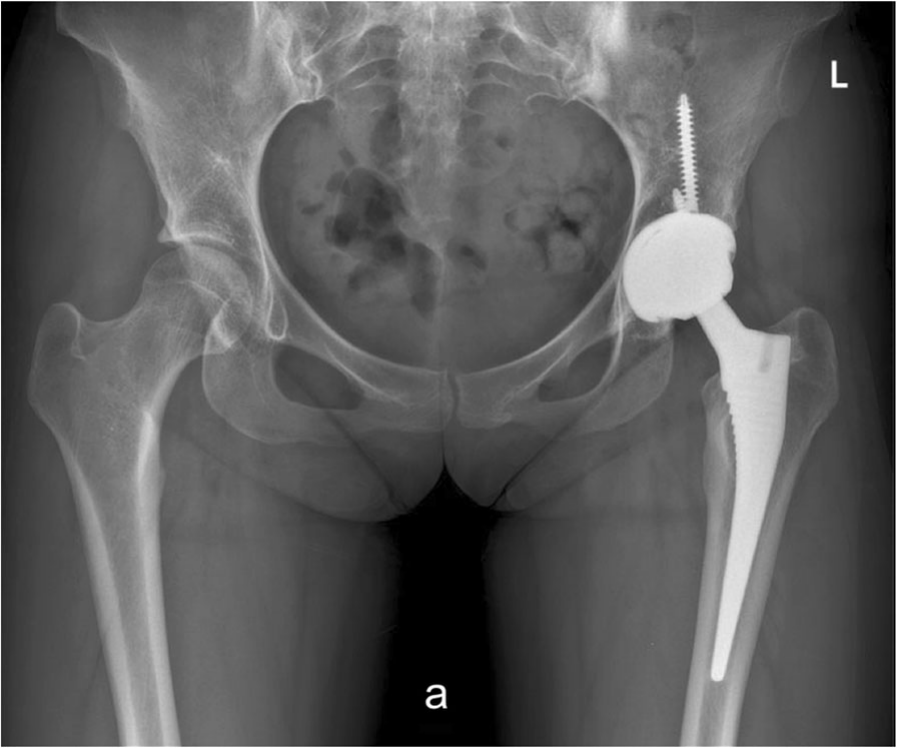
A radiograph was taken 9.1 years after THA showed well-fixed prosthesis (case 16)

The outcomes following cementless CoC THA between groups are presented in Table 3. At the most recent follow-up, there were no clinical or radiological signs of osteolysis or loosing after CoC THA in PVNS patients, which indicated no recurrence of PVNS (Fig. 3). The results of radiologic evaluations by the two different surgeons were consistent. There was no significant difference between the PVNS group and the control group for HHS scores prior to the THA and at the most recent follow-up. In the PVNS group, the mean HHS improved from 48.7 (range 39–62) points preoperatively to 92.6 (range 81–99) at the last follow-up, and was statistically significant (*P* < 0.001). All patients could perform moderate physical activities without crutches, and five patients could perform strenuous activities regularly, including running, sports, and climbing.

**Table 3:** The outcomes following cementless ceramic-on-ceramic total hip arthroplasty between the PVNS group and the control group

	PVNS (*n* = 19)	Controls (*n* = 38)	*p* value
Mean HHS, point, SD			
Preoperative	48.7 ± 3.8	48.2 ± 4.1	0.612
Latest follow-up	92.6 ± 5.5	93.4 ± 4.6	0.584
Aseptic loosening, *n* (%)	1 (5.3%)	1 (2.6%)	0.614
Infection, *n* (%)	0 (0%)	1 (2.6%)	0.480
Osteolysis, *n* (%)	0 (0%)	0 (0%)	–
Dislocation, *n* (%)	0 (0%)	0 (0%)	–
Ceramic fracture, *n* (%)	0 (0%)	0 (0%)	–
Squeaking, *n* (%)	1 (5.3%)	0 (0%)	–
Any revision, *n* (%)	1 (5.3%)	2 (5.3%)	1.000

*PVNS* pigmented villonodular synovitis, *HHS* Harris Hip Score, *SD* standard deviation

**Fig. 4:**
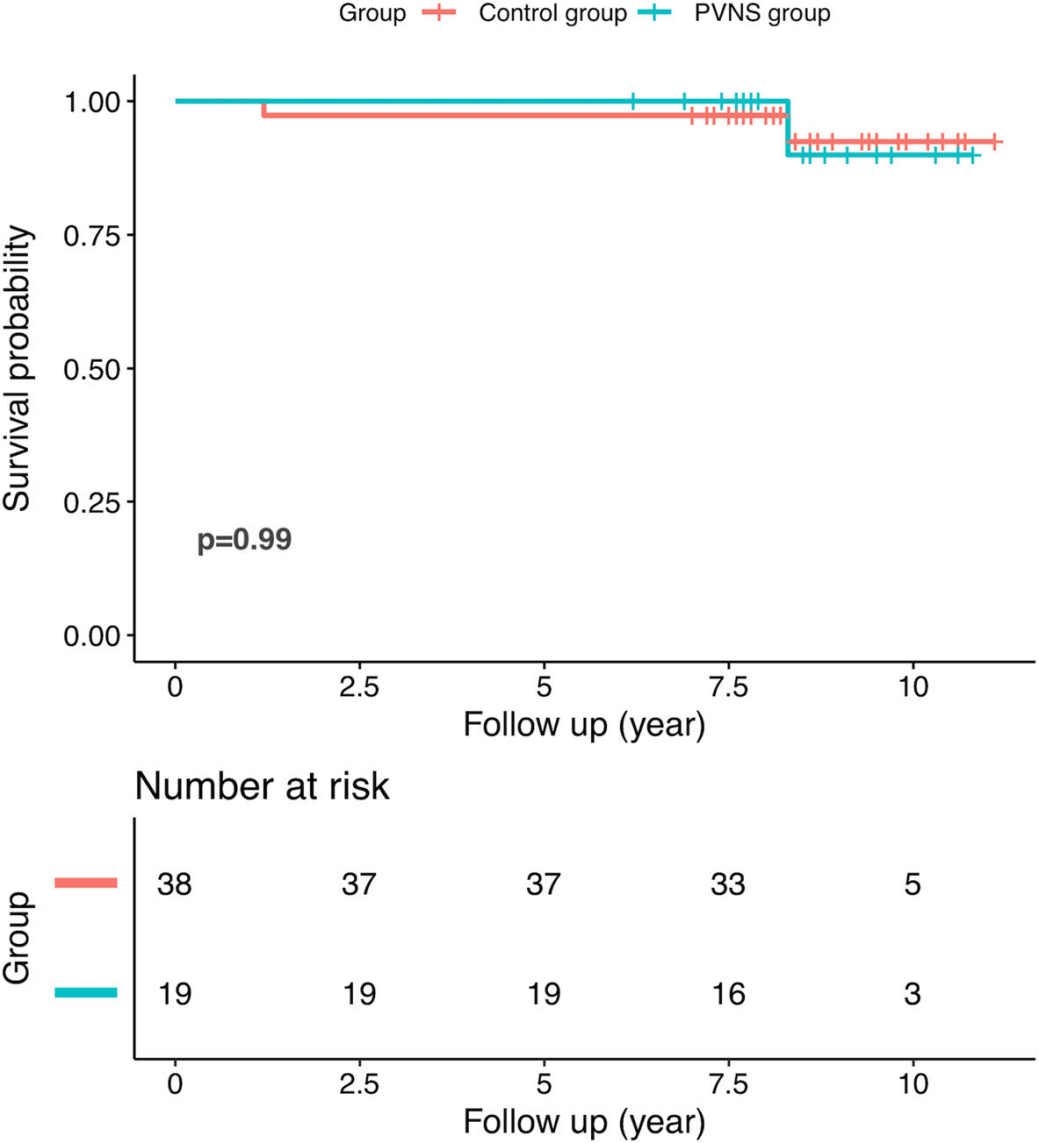
A Kaplan-Meier implant survivorship curve of patients with and without PVNS

One patient (case 2) underwent revision due to aseptic loosening in the PVNS group, while another patient (case 7) developed a squeaking complication 2 years postoperatively, but did not require revision. In the control group, two patients underwent revision (one aseptic revision and one infection) (*p* = 1.000). For the patient who underwent aseptic revision in the PVNS group, no pathological evidence of recurrence of PVNS was found by histological examination. None of the patients experience osteolysis, dislocation, or ceramic fracture in either group. The implant survivorship free of any revision was 90.0% (95% CI, 73.2% to 100%) in the PVNS group and 92.5% (95% CI, 82.6% to 100%) in the control group at 10 years (*p* = 0.99) (Fig. 4).

## Discussion

This study demonstrated that patients with PVNS undergoing cementless CoC THA had similar survivorship and functionality compared to those with the diagnosis of osteonecrosis of femoral heads or osteoarthritis of hips. None of the patients with PVNS experienced infection, osteolysis, dislocation, or ceramic fracture after a mean follow-up of 8.6 years. Moreover, there was no evidence of recurrent PVNS during the middle-term follow-up in the present study.

PVNS is a proliferative disorder characterized by infiltration of hemosiderin-laden macrophages, multinucleated giant cells, and inflammatory cells which carry a high risk of recurrence [22–24]. Due to its low incidence, PVNS is not well understood. While a consensus regarding the pathogenesis of PVNS has not yet been established, some researchers have hypothesized that trauma-induced hemorrhage could be a causative factor [25, 26]; in previously published case series, prior trauma ranged from 3 to 53% [27] In our study, 4 of 19 patients with PVNS (21.1%) had had a history of hip trauma before the diagnosis of PVNS. Although other researchers suggested patients with PVNS commonly have abnormal serum inflammation markers [28], there were only two patients with PVNS (10.5%) with elevated ESR and CRP in our study. However, our results correspond to those reported by Gitelis et al. who initially documented a lack of an inflammatory syndrome associated with PVNS [27].

Despite improved imaging, definitive preoperative diagnosis of PVNS is difficult without histopathology. At the early stage of PVNS, the treatment primarily includes either open or arthroscopic synovectomy. However, PVNS has a high recurrence rate, up to 25%, likely due to incomplete debridement without neck cutting [2]. In the present study, five patients with PVNS had undergone previous arthroscopic synovectomy; the duration between the last synovectomy and the primary THA were 0.8, 2, 2.5, 3.1, and 3.6 years (mean 2.4 years). We hypothesize that the short-term recurrence may be related to incomplete synovectomy by arthroscopy technique.

At end-stage PVNS, THA is the most effective treatment option, with lower PVNS recurrence rates and lower revision rates than synovectomy alone [8]. The lifespan of the primary THA is of significant concern, as patients with PVNS are typically younger and will therefore retain the implants for a longer duration of time. The two most important aspects of implant longevity are the method of implant fixation and the type of bearing surface [14]. While case studies of PVNS of the hip are limited, both cemented and cementless THA have been reported to result in good outcomes [14].

No recurrent PVNS was found during the follow-up in the present study, which is similar to prior studies [5, 7, 13, 14]. Yoo et al. [5] reported eight PVNS patients with cementless THA and none of the patients had clinical or radiographic evidence of recurrent PVNS for an average of 8.9 years follow-up. Recently, Tibbo et al. [7] reviewed 25 patients PVNS who underwent THA with conventional polyethylene or highly crosslinked polyethylene bearings during a mean of 10 years follow-up. Of them, only one patient (4%, 1/25) occurred recurrence at 24 years postoperatively.

Of the studies involving cementless THAs, most of the studies, however, only reported on polyethylene or metal bearing surfaces [5, 7, 14, 27]. Hoberg et al. [29] reported two cases of metal-on-metal hip resurfacing in patients with PVNS. Yoo et al. [6] described revisions due to prosthesis loosening and osteolysis in two of eight patients who had undergone THA using metal-non-cross-linked polyethylene bearing and periprosthetic osteolysis; additionally, two other patients had loosening with zirconia-non-cross-linked polyethylene. The need for revisions and the shorter implant survivorship in these cases is thought to be due to conventional polyethylene induced-wear and metal ion-induced osteolysis resulting in aseptic loosening [28, 29]. Recently, Tibbo et al. reported the 10-year survivorship free from any revision was 100% for highly crosslinked polyethylene liners and 66% for conventional polyethylene liners [8]. The Ceramic-on-ceramic bearing surface, characterized by its superior hardness and smoothness, has the lowest wear rates in comparison to all other bearing surfaces in THA [30, 31]. In our study, only one patient underwent a revision arthroplasty due to aseptic loosening. The 10-year implant survivorship free of any revision after CoC THA was 90.0% in the PVNS group, which was similar to that in the control group (92.5%, *p* = 0.99). Moreover, we did not identify any local recurrence in the PVNS cohort.

There are several limitations in our study. A major limitation was the retrospective case-control design of the study, which is subject to inherent biases of the design. Additionally, we did not sort the disease manifestations (diffuse vs. focal) based on pathological classification, although all cases were diagnosed by histological evaluations. Another limitation is that we cannot compare outcomes of different bearing surfaces, such as metal-on-metal, metal-on-polyethylene, or ceramic-on-polyethylene articulation, as PVNS patients in this study were all treated with CoC THA.

## Conclusions

In conclusion, end-stage PVNS of hip treated with cementless CoC THA had a low complication rate with similar implant survivorship and restoration of function in comparison to other young patients with osteonecrosis or osteoarthritis. These findings indicate that utilization of cementless THA ceramic-on-ceramic bearings could be a viable option for young patients with PVNS.

## Abbreviations

ASA: American Society of Anesthesiologists
CI: Confidence interval
CoC: Ceramic-on-ceramic
CRP: C-reactive protein
ESR: Erythrocyte sedimentation rate
HHS: Harris Hip Scores
PVNS: Pigmented villonodular synovitis
THA: Total hip arthroplasty

## References

[1] Hufeland M, Gesslein M, Perka C, Schröder JH (2018). Long-term outcome of pigmented villonodular synovitis of the hip after joint preserving therapy. Arch Orthop Trauma Surg.

[2] Levy DM, Haughom BD, Nho SJ, Gitelis S (2016). Pigmented Villonodular synovitis of the hip: a systematic review. Am J Orthop.

[3] Startzman A, Collins D, Carreira D (2016). A systematic literature review of synovial chondromatosis and pigmented villonodular synovitis of the hip. Phys Sportsmed.

[4] Lu H, Chen Q, Shen H (2015). Pigmented villonodular synovitis of the elbow with rdial, median and ulnar nerve compression. Int J Clin Exp Pathol.

[5] Byrd JWT, Jones KS, Maiers GP (2013). Two to 10 years’ follow-up of arthroscopic management of pigmented villonodular synovitis in the hip: a case series. Arthroscopy.

[6] Yoo JJ, Kwon YS, Koo K-H, Yoon KS, Min BW, Kim HJ (2010). Cementless Total hip arthroplasty performed in patients with pigmented villonodular synovitis. J Arthroplast.

[7] Li LM, Jeffery J (2011). Exceptionally aggressive pigmented villonodular synovitis of the hip unresponsive to radiotherapy. J Bone Joint Surg Br..

[8] Tibbo ME, Wyles CC, Rose PS, Sim FH, Houdek MT, Taunton MJ (2018). Long-term outcome of hip arthroplasty in the setting of pigmented villonodular synovitis. J Arthroplast.

[9] Atrey A, Wolfstadt JI, Hussain N, Khoshbin A, Ward S, Shahid M (2018). The ideal total hip replacement bearing surface in the young patient: a prospective randomized trial comparing alumina ceramic-on-ceramic with ceramic-on-conventional polyethylene: 15-year follow-up. J Arthroplast.

[10] Swarup Ishaan, Lee Yuo-yu, Chiu Yu-fen, Sutherland Ryan, Shields Marisa, Figgie Mark P. (2018). Implant Survival and Patient-Reported Outcomes After Total Hip Arthroplasty in Young Patients. The Journal of Arthroplasty.

[11] Costi K, Solomon LB, McGee MA, Rickman MS, Howie DW (2017). Advantages in using cemented polished tapered stems when performing total hip arthroplasty in very young patients. J Arthroplast.

[12] Archibeck MJ, Surdam JW, Schultz SC, Junick DW, White RE (2006). Cementless total hip arthroplasty in patients 50 years or younger. J Arthroplast.

[13] Kuo F-C, Liu H-C, Chen W-S, Wang J-W (2012). Ceramic-on-ceramic total hip arthroplasty: incidence and risk factors of bearing surface-related noises in 125 patients. Orthopedics.

[14] Elzohairy Mohamed Mansour (2018). Pigmented villonodular synovitis managed by total synovectomy and cementless total hip arthroplasty. European Journal of Orthopaedic Surgery & Traumatology.

[15] JAFFE H (1941). Pigmented villonodular synovitis, bursitis, and tenosynovitis. Arch Pathol.

[16] Williams VG, Whiteside LA, White SE, McCarthy DS (1997). Fixation of ultrahigh-molecular-weight polyethylene liners to metal-backed acetabular cups. J Arthroplast.

[17] CHARNLEY JOHN, HALLEY DAVID K. (1975). Rate of Wear in Total Hip Replacement. Clinical Orthopaedics and Related Research.

[18] DeLee JG, Charnley J. Radiological demarcation of cemented sockets in total hip replacement. Clin Orthop Relat Res. 1976;121:20–32. 991504

[19] Gruen TA, McNeice GM, Amstutz HC. “Modes of failure” of cemented stem-type femoral components: a radiographic analysis of loosening. Clin Orthop Relat Res. 1979;141:17–27. 477100

[20] Joshi RP, Eftekhar NS, McMahon DJ, Nercessian OA (1998). Osteolysis after Charnley primary low-friction arthroplasty. A comparison of two matched paired groups. J Bone Joint Surg Br.

[21] Smith JH, Pugh DG (1962). Roentgenographic aspects of articular pigmented villonodular synovitis. Am J Roentgenol Radium Therapy, Nucl Med.

[22] Colman MW, Ye J, Weiss KR, Goodman MA, McGough RL (2013). Does combined open and arthroscopic synovectomy for diffuse PVNS of the knee improve recurrence rates?. Clin Orthop Relat Res.

[23] Shoji T, Yasunaga Y, Yamasaki T, Nakamae A, Mori R, Hamanishi M (2015). Transtrochanteric rotational osteotomy combined with intra-articular procedures for pigmented villonodular synovitis of the hip. J Orthop Sci.

[24] Chiari C, Pirich C, Brannath W, Kotz R, Trieb K (2006). What affects the recurrence and clinical outcome of pigmented villonodular synovitis?. Clin Orthop Relat Res.

[25] Ma X, Shi G, Xia C, Liu H, He J, Jin W (2013). Pigmented villonodular synovitis: a retrospective study of seventy five cases (eighty one joints). Int Orthop.

[26] Baba S, Motomura G, Fukushi J, Ikemura S, Sonoda K, Kubo Y (2017). Osteonecrosis of the femoral head associated with pigmented villonodular synovitis. Rheumatol Int.

[27] Steinmetz S, Rougemont A-L, Peter R (2016). Pigmented villonodular synovitis of the hip. EFORT Open Rev.

[28] Xie G, Jiang N, Liang C, Zeng J, Chen Z, Xu Q (2015). Pigmented villonodular synovitis: a retrospective multicenter study of 237 cases. PLoS One.

[29] Hoberg M, Amstutz HC (2010). Metal-on-metal hip resurfacing in patients with pigmented villonodular synovitis: a report of two cases. Orthopedics.

[30] Hannouche D, Devriese F, Delambre J, Zadegan F, Tourabaly I, Sedel L (2016). Ceramic-on-ceramic THA implants in patients younger than 20 years. Clin Orthop Relat Res.

[31] Cai Y, Yan S (2010). Development of ceramic-on-ceramic implants for total hip arthroplasty. Orthop Surg.

